# Aging: A Predisposition to Dry Eyes

**DOI:** 10.1155/2014/781683

**Published:** 2014-08-14

**Authors:** Anushree Sharma, Holly B. Hindman

**Affiliations:** ^1^Flaum Eye Institute, University of Rochester School of Medicine and Dentistry, 601 Elmwood Avenue, Rochester, NY 14642, USA; ^2^Center for Visual Science, University of Rochester School of Medicine and Dentistry, Rochester, NY 14642, USA

## Abstract

Dry eye syndrome is a disease of the ocular surface and tear film that is prevalent in older adults. Even though the degree of visual acuity loss in dry eye patients is commonly mild-to-moderate, in the aging population, this minimal change in visual status can lead to a significant decrease in visual function and quality of life. A healthy ocular surface is maintained by appropriate tear production and tear drainage, and deficiencies in this delicate balance can lead to dryness. In the aging eye, risk factors such as polypharmacy, androgen deficiency, decreased blink rates, and oxidative stress can predispose the patient to developing dry eye that is frequently more severe, has higher economic costs, and leads to worse consequences to the well-being of the patient. Understanding why elderly patients are at higher risk for developing dry eyes can provide insights into the diagnosis and management of the growing number of older adults struggling with dry eye and minimize the burden of disease on our aging population.

## 1. Introduction

Dry eye syndrome, or keratoconjunctivitis sicca, is a multifactorial disease of the tear film and ocular surface resulting in eye discomfort and compromised visual quality. Dysfunction of any component of the lacrimal gland, ocular surface, eyelids, and nervous system can cause dry eyes. Dry eye is especially common in the elderly, occurring in approximately 5–30% of the general elderly population, and affects women more commonly than men [1]. The prevalence disparity by age ranges from 8.4% in subjects younger than 60 years old to 15% in patients 70–79 years old and 20% in those older than 80 years [2, 3]. Various factors predispose older adults to dry eyes including higher rates of systemic and topical medication use, lid laxity, hormonal changes (menopause), inflammatory systemic conditions, and oxidative stress. With greater life expectancy, a growing number of people are expected to join the over-60 age group and the prevalence of dry eye disease is therefore expected to increase even more.

Patients with dry eye experience blurred vision, foreign body sensation, pain, injection, epiphora, and, in severe cases, loss of vision. While high-contrast visual acuity may not be affected or may be only minimally reduced, individuals with dry eye can suffer from discomfort and/or functional vision changes that can be debilitating. In a study assessing the impact of severe dry eye disease (DED) on patients' lives, subjects with severe dry eye reported an impact similar to that reported in other studies for moderate to severe angina or dialysis [4]. Quality of life is affected since even with mild vision loss, such as that which most commonly occurs with DED, the risk of falling is increased 2-fold, the risk of depression is increased 3-fold, and the risk of hip fracture is increased 4-fold [5–7]. Hip fractures, known to cause significant morbidity among the elderly, are more common amongst individuals with lower vision; 8.5% of hip fractures occur in elderly patients with mild-to-moderate vision loss (VA between 20/30 and 20/80) which can result from dry eyes. In contrast, only 3% of hip fractures occur in older patients with a VA of 20/25 or better [8]. Hip fractures can lead to decreased independence, functional status, and quality of life.

Along with the physiologic toll on patients, DED also generates a significant economic burden on this population. A study by Yu et al. determined the average annual direct medical cost per DED patient to be $678 for mild dry eye, $771 for moderate dry eye, and $1267 for severe dry eye [9]. In light of higher DED prevalence in patients over 50 years of age, this amounts to $3.84 billion for the health care system to support cost of ocular lubricants, cyclosporine, punctal plugs, nutritional supplements, and professional healthcare visits as well as loss of workplace productivity. In indirect costs, the average annual cost to society per patient was $11,302 and the overall societal burden was $55.4 billion [9].

It is important to bear in mind that even seemingly benign impairments from dry eyes, such as impaired reading, can lead to errors in medication administration [10] which can be life-threatening. Quality of life can be severely affected—with DED being likened to chronic pain syndromes [11], being associated with poorer general health [12], and leading to greater problems in daily activities by factors 2-3x over those of normal [13]. In light of the susceptible state of the aging population for developing dry eyes, early screening, prompt attention, and targeted cost-effective treatment can make a difference in a patient's mental health, self-confidence [14], and functional status.

## 2. Pathophysiology of Dry Eyes and Implications for the Aging Eye

The tear film consists of the following 3 main layers: the lipid layer secreted by the meibomian glands, the aqueous layer produced by the lacrimal gland, and the mucin layer secreted by conjunctival goblet cells. Homeostasis of the ocular surface environment is maintained by a fine balance of tear production and appropriate tear drainage. Tear production is orchestrated by multiple components including the lacrimal gland that secretes the aqueous component of the tears, which comprises the thickest layer of the tear, and the corneal nerves which provide a reflex loop that modulates tear production in response to different conditions [15]. Appropriate tear drainage is maintained by the physical apposition of the eyelids to the globe that minimizes tear evaporation, meibomian glands that contribute to tear-film stability [16, 17], the blink mechanism that adequately distributes the tear film across ocular surface, and the orbicularis muscle that drives the flow of tears medially, maintains the thickness of the tear film over the cornea, and directs closure of the lacrimal punctum [18]. The Dry Eye Workshop (DEWS) classification divides dry eye into two major pathophysiologic categories—aqueous tear deficiency and evaporative dry eye—with older adults being more susceptible to both categories [19].

## 3. Aqueous Tear Deficiency Changes with Aging

Decreased tear production as a consequence of lacrimal gland dysfunction, altered reflex secretion, diminished corneal sensation, or inflammatory destruction of lacrimal glands lead to tear deficiency—a major cause of dry eye. Older adults are particularly susceptible to inadequate tear production because they have a higher prevalence of autoimmune diseases (Sjögren's syndrome and rheumatoid arthritis), decreased corneal sensitivity, and medicamentosa (polypharmacy), which contribute to the etiologic mechanisms and can, in severe cases, have vision threatening consequences. Inadequate aqueous tear film drives tear hyperosmolarity that induces an inflammatory cascade, leaving epithelial cells dead or devitalized [20]. Goblet cells produce mucin which bears a protective function by clearing debris, preventing bacterial adhesion, promoting boundary lubrication, and maintaining the epithelial barrier function [21]. In the setting of inflammation, the goblet cell number and secretory function are decreased and inflammatory cytokines such as interferon gamma and TNF alpha induce goblet cell apoptosis—further diminishing mucin production [22–24]. Corneal epithelial cells with inadequate mucin protection are left vulnerable to cell damage. The loss of goblet cells from injury induced by the inflammatory response further perpetuates the loss of corneal epithelial cells. A spectrum of ocular surface disease can then result—ranging from mild dry eye, to particularly painful symptoms associated with filament and mucous plaque formation. With aging, the number of goblet cells remains unchanged; however, the cell functions decline [25]; in addition, the aging conjunctival cells are more prone to apoptosis [26]. In the setting of dry eyes in older adults, a cumulative higher loss of functional goblet cells and increased level of goblet cell apoptosis occur which can lead to advanced DED. At the advanced stages of the disease spectrum, corneal keratinization, corneal ulceration, and band keratopathy can arise from repeated or severe insults over time.

Systemic and topical medications are a key risk factor predisposing the over-60 population to sicca from deficient tear production. The CDC reported that greater than 76% of Americans 60 years or older used two or more prescription drugs and 37% used five or more between 2007 and 2008. Only 10.8% of younger adults (18–44 year olds) take 5 or more drugs, whereas 41.7% of middle aged adults and up to 47.5% of older adults take 5 or more medications [27, 28]. Systemic medications including antidepressants, diuretics, dopaminergic drugs for Parkinson's disease, and antimetabolites frequently used in treating rheumatoid arthritis are all prescription drugs that cause or exacerbate dry eyes and are used commonly in older patients. Drug clearance also changes with aging as hepatic and renal function decline. Reduced clearance of drugs such as diuretics [29] leads to increased plasma half-life and increased sensitivity to drugs [30]. The Beaver Dam Eye Study found an overall 10-year dry eye incidence of 21.6% among individuals aged from 43 to 84 years, with an increase in incidence from 17.3% in subjects in the 48- to 59-year-old age group, to 28.0% in those 80 years or older [31]. This study also showed that patients using decongestants, antihistamine, and vitamins have a higher incidence of dry eyes [31]. With increasing use of such over-the-counter (OTC) medications and supplements in the elderly, the prevalence of medication induced dry eye is expected to be even higher than that among younger age groups. In the elderly, the underlying systemic disease for which they are taking systemic medications is often more severe or has persisted for a longer duration, making the long-term use of dry eye-inducing systemic medications more likely and increasing the likelihood of developing medication-induced sicca. Dry eye, for example, is frequent in patients using antidepressant medications. Older patients are particularly at risk for developing antidepressant-induced dry eye because they tend to have longer duration of depression and take antidepressant medications for a longer period of time [32].

The use of topical medications, such as topical glaucoma medications, can also increase the risk of development of dry eye relative to age matched controls [33]. Glaucoma is more prevalent in the elderly [34], with increasing numbers of people on more than one topical medication. Sixty-one percent of patients using one or more pressure lowering drops have decreased tear production (<5 mm) on Schirmer's test indicating tear deficiency dry eye [35]. In adults using glaucoma drops, 63% developed signs and symptoms consistent with dry eye and did so at a mean age of 55. In patients not using glaucoma drops, only 23% developed symptoms and signs of dry eye but did not do so until a mean age of 70 [36]. Earlier disease onset translates into a longer course of ocular discomfort, higher cumulative healthcare burden, and more severe morbidity. Ocular surface disease due to glaucoma medications correlates with the number of medications containing preservatives such as benzalkonium chloride (BAK), which can cause tear film instability, loss of goblet cells, conjunctival squamous metaplasia and apoptosis, disruption of the corneal epithelium barrier, and damage to deeper ocular tissues even at low concentrations [37]. More severe ocular surface disease has also been seen with preservative containing eye drops. Schwab et al. showed that long-term glaucoma medications with preservatives can cause conjunctival foreshortening with shrinkage including conjunctival scarring [38, 39]. Furthermore, a significant foreshortening of the inferior conjunctival fornix was found with aging independent of medication use [38]. Since more elderly patients use glaucoma eye drops, many of which contain preservatives, the older adults on glaucoma medications with preservatives are at an even increased risk of severe dry eye sequelae.

Another common disease causing aqueous dry eye in the elderly, especially older women, is lacrimal gland dysfunction. Notably, older men and women are almost twice as likely to have dry eyes compared with their younger counterparts. The dry eye prevalence is 3.90% among men aged from 50 to 54 years compared to 7.67% among men 80 years and older [40]. Similarly, dry eye prevalence is 9.8% among women aged 75 years or older compared to only 5.7% among women less than 50 years old [41]. Secretory function of the lacrimal gland is known to be regulated by androgens [42, 43]. Dehydroepiandrosterone sulphate (DHEAS) is one of the main adrenal androgens. Serum levels of DHEAS are lower in women with Sjögren's syndrome, older men, and older women [44]. Decreased DHEAS levels in older men correlate with dry eye symptoms and decreased Schirmer's test (<5 mm) due to insufficient lacrimal gland function; however, the association is weak (*r* = 0.13) [45]. Since women have lower levels of androgens compared to the levels in men, further age-related decreases in androgen levels may diminish the androgen levels below the critical amount needed for optimum eye health [46]. Along with a decrease in androgen levels, postmenopausal women develop lower levels of estrogen—a hormone that is known to stimulate meibomian glands and help regulate ocular surface homeostasis [42]. Together, androgen deficiency and estrogen decrease lead to inadequate lacrimal gland secretion with superimposed tear-film instability in older women and higher risk of developing dry eye.

## 4. Tear Evaporation Changes with Aging

The second major component of dry eye pathophysiology is the rate of tear film evaporation. Multiple features are at play to effectively conserve tears over the ocular surface. Eyelid apposition to the globe minimizes exposure, appropriate frequency of blinking ensures constant renewal of the tear film across the corneal surface, lipid production from meibomian glands stabilizes the tear film, and the orbicularis portion of the eyelid directs tear outflow at a controlled rate [47]. Abnormalities in eyelid positioning (laxity, floppy eyelid syndrome, retraction, and lagophthalmos), meibomian gland dysfunction, rosacea, abnormal corneal sensation, and decreased blink reflex are significant contributors to rapid tear film break-up and are seen increasingly in older adults [48–50]. Horizontal lid laxity, for example, is notable in elderly patients and is the most common cause of involutional eyelid malposition. Eyelid malposition, in turn, leads to corneal exposure, poor tear-film distribution, and abnormal tear outflow that induce sicca. Prevalence of involutional entropion in patients 60 or older has been reported as 2.1% and of involutional ectropion as 2.9% [48]. Patients with malpositioned lids can subsequently develop chronic blepharitis, chronic conjunctivitis, superficial punctate keratopathy from abnormal meibomian gland secretory function, mechanical injury, and exposure. As many as 50–70% of patients with malpositioned lids develop dry eye syndrome [48].

Conjunctivochalasis is another notable contributor to poor tear outflow and is characterized by a redundant bulbar conjunctiva interposed between the globe and the eyelid [51]. The prevalence of conjunctivochalasis increases dramatically with age from less than 71.5% in patients 50 years or younger, to greater than 98% in patients above 61 years of age [52]. Pathogenesis of conjunctivochalasis is under investigation; however, elastotic degeneration from cumulative sun exposure and inflammatory degeneration from delayed tear film clearance have been proposed [51, 53, 54]. Once formed, the redundant folds interfere with the inferior tear meniscus and, in some cases, cause occlusion of the inferior punctum. Elderly patients often have higher collective sun exposure that can predispose to development of conjunctivochalasis and often have aqueous tear deficiency that can be exacerbated by disruption of the tear meniscus. Older adults with coexisting eyelid malpositioning can have worsening of appropriate tear flow medially that can cause further pooling of inflammatory cytokines and reinforce conjunctival degeneration.

## 5. Corneal Sensitivity Changes with Aging

A gradual reduction in corneal sensitivity has been shown to occur with increasing age that predisposes older adults to dry eyes [50]. Roszkowska et al. reported that mechanical sensitivity of peripheral cornea decreases gradually throughout life, whereas central corneal sensitivity remains stable until age 60 and then decreases sharply subsequently [55]. The role of corneal sensitivity in dry eye patients, however, is conflicting. Some studies show decreased sensitivity in dry eyes compared with controls on noncontact esthesiometer measurements [56, 57]. Other groups demonstrate hypersensitivity in patients with dry eyes that may result from compromised epithelial barrier function [58, 59]. A characteristic change in dry eye patients that is generally agreed upon is a beadlike transformation of the nerves that is thought to represent nerve damage due to inflammatory processes in DED [60, 61]. Regardless of the direction of change in corneal sensitivity, elderly patients with DED are placed at higher risk of developing sicca signs and symptoms due to corneal nerve alterations. Older adults with corneal hypersensitivity experience increased ocular surface discomfort, while those with decreased sensitivity are prone to complications of exposure keratopathy.

## 6. Neurodegenerative Disease Contributes to Dry Eye among Older Adults

Neurodegenerative diseases can also predispose the aging population to evaporative dry eye. Parkinson's disease, for example, has an incidence of 13.4 per 100,000; only 4% of the cases occur in patients younger than 50 years [62]. Patients with Parkinson's disease have lower blink rates compared with controls [63]. In Parkinson's patients, dopaminergic dysfunction is thought to play a role in decreasing the blink reflex that leads to dry eyes. In addition to lower blink rates, Parkinson's patients with DED also exhibit decreased corneal sensation. Mean corneal sensitivity is shown to be decreased in some studies even in otherwise healthy patients with DED and correlates negatively with age [64]. The decreased corneal sensitivity exacerbates the risk of exposure keratopathy in DED patients and can be especially severe in a patient with a neurodegenerative disease. Parkinson's disease patients, for example, are relatively asymptomatic when compared to sicca counterparts without neurodegenerative disease. These patients may be brought in for evaluation when their family members identify a significant decline in visual function or obvious ocular change. Despite being relatively asymptomatic, these patients frequently have exam findings consistent with the advanced sequelae of chronic dry eye.

## 7. Inflammation and Oxidative Stress

Inflammation and oxidative stress, which increase in aging [65], may play a key role in dry eye development in the elderly. Increased levels of osmolarity and inflammatory cytokines have been detected in the tears of dry eye patients [66]. Tear concentrations of IL-6, IL-8, and TNF-*α* have been shown to be significantly higher in DED and can further amplify inflammation by recruiting activated immune cells [67]. Other inflammatory markers, such as IFN-*γ*, can promote goblet cell loss and stimulate keratinization of conjunctival epithelium [68]. Healthy adults acquire a baseline chronic low-grade inflammation with advancing age that is precipitated by constant antigenic load [69]. Older adults with DED, hence, bear damage via inflammatory cytokines from normal aging as well as from sicca. Oxidative stress, a counterpart of inflammation, occurs when antioxidants are unable to counteract reactive oxygen species (ROS) that are generated in normal metabolic processes. Production of aggressive oxygen species such as free radicals and peroxides leads to DNA damage over time, inducing cell necrosis. In younger, healthy human bodies, low levels of ROS are counteracted by antioxidant enzymes. With accumulation of radical species over time such as that which occurs in aging, oxidative stress activates cell regulatory pathways that can alter the regenerative capacity of cells such as the corneal epithelial cell layer under dry eye conditions [65]. Furthermore, inflammatory conditions such as rosacea and blepharitis, commonly seen in the aging population, are characterized by release of cytokines and chemokines which can induce further free radical production [70, 71]. Poorly healing epithelium in the setting of inflammation in the elderly can thus cascade into severe corneal conditions such as erosions, keratitis, or ulcers.

## 8. Treatment from the Aging Perspective

Standard of care therapy with artificial tears, cyclosporine, punctal plugs, steroids, or antibiotics can be effective in the elderly population. For mild dry eye, lubrication with artificial tear drops and gels is a key initial step to address patient symptoms. Environmental factors contributing to tear deficiency or evaporation should be minimized. Cigarette smoking, for example, has been found to adversely affect the lipid layer of the tear film and thus can negatively affect tear-film stability [72]. Dry eye inducing medications such as antihistamines or diuretics should be avoided if possible, and evaporative forces such as air conditioners or ceiling fans and low-humidity environments should be minimized [73]. For mild-to-moderate dry eye, treating the underlying diseases—blepharitis, rosacea, autoimmune disease, and others—with topical or oral antibiotics, low-dose steroids, warm compresses, or lid hygiene is necessary. Cyclosporine is an immunosuppressive that inhibits T-cell activation [74] and can be effective in cases of dry eye associated with inflammation [75]. Low dose corticosteroids can help decrease ocular irritation symptoms, decrease corneal fluorescein staining, and improve filamentary keratitis [76].

Punctal plugs can be efficient means to decrease the drainage of tears and prolong the retention time of tears on the ocular surface. They can, thus, improve tear volume and have similar efficacy with upper or lower tear duct occlusion [77]. In severe dry eyes, oral cholinergic agonists such as pilocarpine and cevimeline may improve symptoms by stimulating lacrimal gland secretion [78, 79]. Autologous serum drops have been reported to improve ocular irritation symptoms as well as conjunctival and corneal dye staining in patients with Sjögren's syndrome [80]. Though the role of hormone replacement therapy in dry eye treatment remains controversial [81–83], postmenopausal women with dry eyes may benefit from phytoestrogen supplementation. Phytoestrogen is a naturally occurring nonsteroidal with estrogenic effects that has been shown to decrease tear osmolarity and improve tear production [84]. Additionally, androgen supplementation for older women may help stimulate lacrimal gland function and reduce ocular discomfort. In a pilot study by Nanavaty et al., androgen patching for 3 weeks resulted in an increase in tear-film break-up time and improved Schirmer's test scores [85]. In addition, all 14 subjects reported improvement in “painful or sore eye” symptom.

A few caveats, however, are warranted for the aging population. Many of the artificial tear products, though affordable, contain preservatives, including BAK. With increased susceptibility to tear film instability, loss of goblet cells, and poor epithelial healing from oxidative stress, the addition of toxic ingredients can potentially exacerbate patient symptoms. Preservative-free artificial tears may therefore be better alternatives for older adults but do come at an increased cost. Other treatment options can also be expensive; punctal plugs plus cyclosporine had the highest annual direct expenditure in the study by Yu et al.—with costs close to 3 thousand dollars [9, 73]. Additionally, cyclosporine use has been associated with ocular burning in 17% of the patients [48] and eyelid malposition may alter the efficacy of punctal plugs if poor globe apposition, lid ectropion, or orbicularis weakness is present. Furthermore, in patients with underlying inflammatory conditions, delayed tear clearance can lead to accumulation of significantly high concentrations of inflammatory cytokines, such as interleukin 1, which can exacerbate epithelial cell damage and goblet cell loss [22, 86]. Ongoing advances in tear substitutes and secretagogues may be especially helpful in this population in addressing specific deficiencies of tear components induced by polypharmacy, menopause, or inflammatory disease. Combination therapy in severe disease may be necessary, especially if the patients are predisposed to microbial keratitis such as in patients with high risk nosocomial infections (frequent hospitalizations, nursing home residents, and diabetics) or exposure keratopathy. In summary, treatments must be individualized to most effectively target the disease process while matching the needs and the resources of the patient. Treatment strategies should be developed in partnership with the patient. It is helpful to communicate to the patient that cures are rare and that your goal is to engage them in developing a management strategy that will work for them.
